# Structurally colored coating films with tunable iridescence fabricated *via* cathodic electrophoretic deposition of silica particles†

**DOI:** 10.1039/c8ra01215f

**Published:** 2018-03-16

**Authors:** Kiyofumi Katagiri, Kensuke Uemura, Ryo Uesugi, Kei Inumaru, Takahiro Seki, Yukikazu Takeoka

**Affiliations:** Department of Applied Chemistry, Graduate School of Engineering, Hiroshima University 1-4-1 Kagamiyama Higashi-Hiroshima 739-8527 Japan kktgr@hiroshima-u.ac.jp; Department of Molecular and Macromolecular Chemistry, Graduate School of Engineering Furo-cho, Chikusa-ku Nagoya 464-8603 Japan ytakeoka@apchem.nagoya-u.ac.jp

## Abstract

In recent years, colloidal arrays of submicrometer-sized monodisperse particles used as structurally colored coatings have drawn great attention due to their non-bleaching properties and low impact on human health and the environment. In this paper, structurally colored coating films were fabricated using monodisperse SiO_2_ particles *via* the cathodic electrophoretic deposition (EPD) technique. The addition of a strong polycation, poly(diallyldimethylammonium chloride) (PDDA), enables the cathodic EPD of SiO_2_ particles and carbon black (CB) additives. Optimizing the quantities of PDDA and CB results in the appearance of vivid structural color from the coating films. The arrangement of the particle array is controllable by varying the pH of the water added to the coating sols for EPD. Structurally colored coating films with and without iridescence, *i.e.*, angular dependence, can be fabricated on demand by a simple operation of the EPD process. In addition, the coating film prepared by cathodic EPD displayed high abrasion resistance because PDDA acts not only as a charge control agent but also as a binder.

## Abstract 2

Structurally colored coatings with and without iridescence can be fabricated by varying pH of coating sols for cathodic electrophoretic deposition.

## Graphical abstract

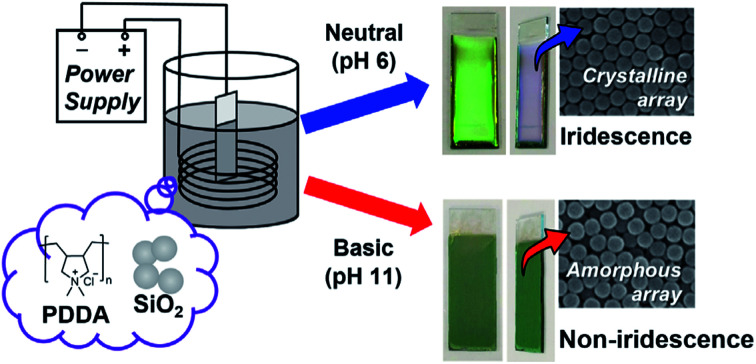

## 1. Introduction

Color plays a significant role in our lives, not only for aesthetics and pleasure but also for communication, signaling, and security. Conventional colored paints produced from organic dyes have an inherent property: they are materials that depend on the dye's chemical nature.^1,2^ Organic dye molecules readily fade over time or upon exposure to light because their coloration mechanism involves the absorption of light based on interactions at a molecular level.^3,4^ On the other hand, inorganic pigments have durability in terms of the fading of their color; however, many inorganic pigments contain heavy metals.^5^ For example, upon combining with other elements, lead forms various colorful pigments, which are widely used in different areas. In recent years, specific concerns have been raised about inorganic pigments due to the hazards of heavy metals to human health and the environment.^5,6^ Therefore, the development of heavy metal-free colored materials with nonfading properties has continuously attracted much attention in the field of colored paints. In fact, numerous objects in nature exhibit colors due to wavelength-specific optical interference, despite their lack of light-absorbing dyes and pigments.^7–15^ The origin of colors can be roughly divided into two categories: some colors are due to absorption of dyes or pigments, and the rest are due to the structure of the material – the latter case is called structural color. Naturally formed precious opals are one of the most familiar naturally occurring examples of structural color, which is a consequence of Bragg diffraction from their ordered internal arrangement of SiO_2_ particles.^16^ Such a structure can be prepared artificially *via* the self-assembly of monodisperse particles as colloidal crystals in which the particle spacing is on the order of the wavelength of light.^17–22^ These colors vary with the viewing angle since the resonance condition changes as the incident light direction varies with respect to the crystal orientation. On the other hand, angle-independent structural color morphologies can also be found in nature.^23–26^ For example, the blue color of avian feathers is caused by the constructive interference of light by submicrometer-sized fine air cavities through spatially distributed scattering.^23,24^ A number of other living things display angle-independent structural colors that originate in a similar manner.^25,26^ Several research groups have attempted to create angle-independent structural colored materials *via* the formation of colloidal assemblies possessing short-range ordered and long-range random (*i.e.*, amorphous) microstructures.^27–39^ Structural color with iridescence is applicable to ornamental jewelry and accessories due to its beautiful appearance.^21^ On the other hand, angle-independent, non-iridescent structural color is favorable for applications that require broad viewing angles, such as building skins, display boards, print media, traffic signals, and colorimetric sensors.^34^ Simple evaporation, thermal-assisted self-assembly, centrifugation, drop-casting, spin-coating, and spray coating techniques have been commonly employed to prepare structurally colored coating films composed of colloidal arrays. The thickness of coating films is not easy to control *via* such techniques. In addition, it is also difficult to coat over surfaces with large-area and/or complicated shapes. Recently, we have successfully prepared colorful coatings consisting of an array of SiO_2_ particles *via* the electrophoretic deposition (EPD) method.^40^ In the EPD process, a DC voltage is applied between a coating substrate and a counter electrode immersed in a particle dispersion.^41–45^ The charged particles dispersed in the polar solvent are attracted by the Coulomb forces to the oppositely charged coating substrate, where they assemble and form a film. EPD offers the benefits of allowing coating with strictly controlled thickness for large area in a much shorter time than other coating techniques. Various vividly colored coatings can be produced from SiO_2_ particles with diameters between 200 nm and 300 nm. In addition, coatings on materials with curved surfaces and complicated shapes, *e.g.*, stainless steel forks, were also successfully prepared *via* the EPD process.^40^ The EPD coating performance strongly depends on the particle surface chemistry. Usually, SiO_2_ particles are negatively charged in aqueous media at pH above their isoelectric point (2.5).^46^ Although strongly acidic conditions can yield positively charged SiO_2_ particles, the charge in such a case is not very large. Therefore, anodic EPD was selected for preparing coating films composed of an array of SiO_2_ particles. However, anodic EPD has some disadvantages; *e.g.*, metal ions are eluted from the coating substrate when metallic substrates are used for the EPD coating.^44^ Such problems are avoidable by applying cathodic EPD.^47–51^ In this case, the cathodic reaction in the EPD process does not cause the elution of metal ions.

The objective of this study is to prepare structurally colored coating films composed of an array of SiO_2_ particles *via* the cathodic EPD process. As mentioned above, SiO_2_ particles can be positively charged only under highly acidic condition (<pH 2). Obtaining positively charged SiO_2_ particles only by controlling pH of the coating sols is not desirable since there is a particular concern regarding the corrosion of coating substrates such as metal plates. Therefore, a wide variety of additives have been used to control the charge of inorganic particles in dispersions. Organic polymers can be utilized to induce steric stabilization, where the polymers are attached to the particle surface, or depletion stabilization, in which the polymers are free in a suspension. Cationic polyelectrolytes (polycations) are widely employed as additives that can invert the surface charge of SiO_2_ particles in a dispersion from negative to positive.^47^ Tatsumisago and Minami successfully prepared thick SiO_2_ coating films *via* cathodic EPD of SiO_2_ particles using a polycation.^50^ The interactions of polycations with SiO_2_ particles were investigated in detail. Baklouti and co-workers reported that the interaction between polycations and the surface of inorganic particles was affected by various factors, such as the nature of the polyelectrolyte, distribution and nature of the oxide surface sites, and properties of the medium.^52^ Therefore, the surface chemistry, including the charge density, of SiO_2_ particles is tunable by varying the characteristics of the polycation additives. Such surface chemistry is expected to affect the arrangement of SiO_2_ particles in the coating films prepared *via* cathodic EPD. It would enable us to easily prepare structurally colored coating films with and without iridescence separately by a simple process. Moreover, adding polycations with inherent binding properties can contribute to enhancing the mechanical stability of the coating films. Conventional EPD coating films prepared from only SiO_2_ particles have poor abrasion resistance because both particle–particle and particle–substrate interactions are very weak. For example, the structurally colored coatings prepared *via* anodic EPD in our previous report^40^ were also very fragile and easy to peel off. Enhancing abrasion resistance is also an important issue for the practical applications of structurally colored coatings.

Herein, we seek to use cathodic EPD to prepare structurally colored coating films. The influences of the cathodic EPD conditions, including the quantity of polycations, on film formation were investigated in detail. The effects of the addition of black additives and the particle diameter on the appearance of structural color were also studied. The color can be tuned simply by adjusting the size of the SiO_2_ particles. In addition, we have examined the control of the packing state of the SiO_2_ particles by tuning the EPD conditions. The resulting structural color of the films, including their texture, would be fundamentally different based on packing state. Both iridescent and non-iridescent structural colors can be controllably prepared by varying the EPD conditions. Finally, the abrasion resistance of the coating films obtained *via* cathodic EPD using polycations has been investigated and compared with those obtained *via* anodic EPD.

## 2. Experimental procedure

### 2.1. Materials

**Fig. 1:**
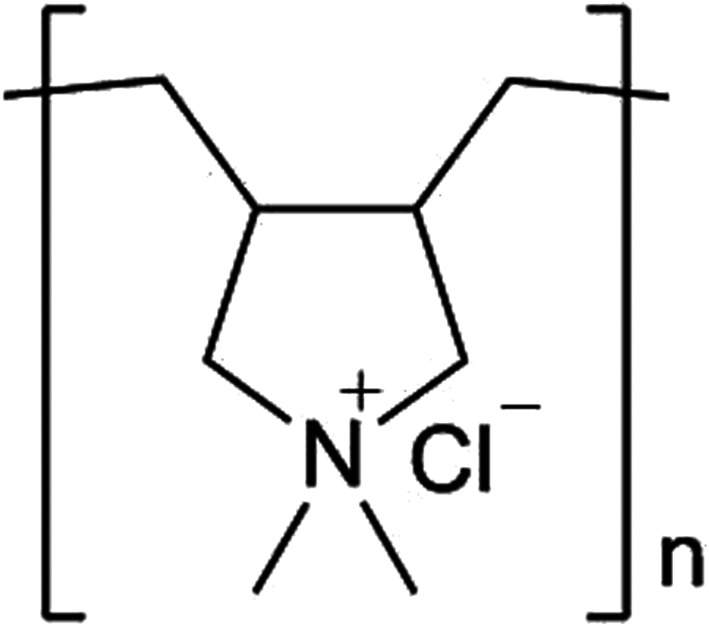
Chemical structure of PDDA.

SiO_2_ particles employed in this work were purchased from Fuji Kagaku Corp., Osaka, Japan. The diameters of particles were 200, 240, 260, 300 and 350 nm. Each of the particles has a narrow size distribution. Carbon black (CB) was gifted from Tokai Carbon Co., Ltd., Tokyo, Japan. The diameters of the CB particles were *ca.* 110 nm. poly(dimethyldiallylammonium chloride) (PDDA, average molar mass: 100 000–200 000 g mol^−1^, Fig. 1) was purchased from Sigma-Aldrich Co., LLC. (St. Louis, MO, USA). Ammonium hydroxide solution (NH_4_OH; 28 wt%), nitric acid (HNO_3_), and ethanol were obtained from Nacalai Tesque Inc., Kyoto, Japan. Water was purified using a Direct-Q UV water purification system (Millipore Corp., Billerica, MA, USA). All other chemicals used in this study were purchased at the highest purity and were used as received.

### 2.2. Cathodic electrophoretic deposition

SiO_2_ and CB particle dispersions for electrophoretic deposition (coating sols) were prepared as follows. SiO_2_ particles were dispersed in water with stirring. NH_4_OH and HNO_3_ were employed for adjusting the pH of the water. The dispersions were placed in an ultrasonic water bath to disperse the particles homogeneously, and ethanol was added after the SiO_2_ particles were dispersed completely. The molar ratio of H_2_O to ethanol was 1 : 4. The amount of SiO_2_ particles added into the dispersions was 1 wt%, and the total weight of the dispersions was 70 g. The coating sol was obtained by adding certain quantities of PDDA and CB to the dispersion. ITO-coated glass plates were employed as the coating substrates. Stainless steel spirals (SUS304) were employed as the counter electrodes. A schematic illustration of the EPD process is shown in Fig. S1 (ESI†). The ITO-coated glass substrates were cleaned using the RCA protocol (*i.e.*, by immersion in a 5 : 1 : 1 H_2_O/H_2_O_2_ aq./NH_4_OH aq. (v/v/v) mixture for 30 min at 60 °C), followed by rinsing with deionized water. The stainless steel spirals were cleaned using ethanol. The ITO-coated glass substrates and the stainless steel spirals were immersed in the coating sols after cleaning. A constant DC voltage was applied between the coating substrate and the spiral using a power supply (PAN110-3A, Kikusui Electronics Corp., Yokohama, Japan), which induced an electrophoretic force on the positively charged SiO_2_ and CB particles toward the cathode substrate, *i.e.*, the ITO-coated glass substrate. After electrophoresis, the coated substrates were withdrawn from the sols at a constant speed (2 mm s^−1^) and dried at room temperature in air.

### 2.3. Characterization

Photographs showing the colors of the coatings were acquired using a digital camera. The arrangement of the SiO_2_ particles in the coating films was investigated using an SEM (Hitachi, S-4800). The samples were coated with a Pt layer using a sputtering apparatus, and the images were obtained with an SEM at 15 kV. The coating substrate was cut at a position 5 mm from the bottom of the substrate for cross-sectional SEM observations (see Fig. S2 (ESI†)). The coating films were fixed with epoxy resin (Epok 812, Okenshoji Co., Ltd., Tokyo, Japan) before the substrate was cut. 2D FFT images were obtained using image analysis software (ImageJ), and a UV-Vis spectrometer (JASCO V-670) was used to measure the relative reflectance and transmission spectra of the coating films. The spectra were acquired on the center position of the coating films. An absolute reflectance measurement unit (ARMN-735) was employed for measuring the relative reflectance spectra. The abrasion test was carried out on sandpaper as an abrasion surface. The coating films were placed against sandpaper (#600 mesh) and weighted with 200 g of weight. The sample was moved 3 cm in a straight manner, and one cycle was finished.

## 3. Results and discussion

### 3.1. Influence of the quantity of polycation on film color

Several types of polycations have been employed during the cathodic EPD of inorganic particles. Polycations with inherent binding properties, such as poly(allylamine hydrochloride) (PAH), polyethylenimine (PEI), and PDDA, could be used for endowing SiO_2_ particles with charge.^47^ PAH and PEI are weak polyelectrolytes, for which the degree of dissociation of the ionic groups depends on the conditions. In contrast, PDDA, which features quaternary ammonium groups, is a strong polyelectrolyte, for which the degree of dissociation of the ionic groups is nearly pH independent over a wide pH range. PDDA can maintain a positive charge even under strongly basic conditions. Therefore, PDDA can be fully charged in solution and adsorb onto the surface of SiO_2_ and CB particles stably. We employed PDDA as a polycationic additive for the experiments in this study.

**Fig. 2:**
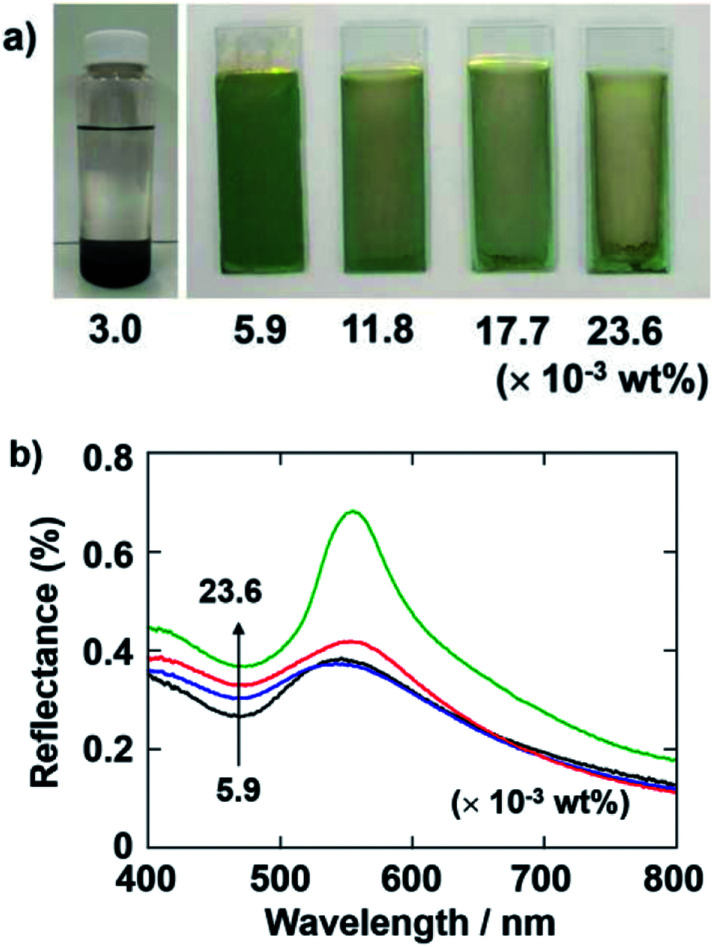
(a) Optical photographs of coating films prepared *via* cathodic EPD using PDDA. The quantity of PDDA added to the coating sol was varied from 3.0 to 23.6 × 10^−3^ wt% SiO_2_ particles with a diameter of 260 nm. The quantity of CB in the coating sols was 3.6 × 10^−3^ wt%. The pH of the water added to the coating sol was 11.3. The applied voltage and deposition time were 7 V and 8 min, respectively. In the case of 3.0 × 10^−3^ wt% PDDA, photographs of the coating sol are given instead of images of the coating films. (b) Reflectance spectra of the coating films shown in (a).

First, we investigated the influence of the concentration of PDDA on the formation of the coating films *via* cathodic EPD. Fig. 2a shows optical photographs of cathodic EPD-coated films on ITO-coated glass substrates prepared from coating sols with various quantities of PDDA, *i.e.*, 3.0–23.6 × 10^−3^ wt% (in the case of 3.0 × 10^−3^ wt%, a photograph of the sol is given). SiO_2_ particles with a diameter of 260 nm were used. CB particles were added to the coating sol to enhance the structural color by reducing the contribution of incoherent light scattering.^31,32^ Here, the quantity of CB in the coating sols was 3.6 × 10^−3^ wt%. To promote the dissociation of the Si–OH groups on the SiO_2_ particles, the pH of the water added to the coating was adjusted to 11.3. The zeta-potential of SiO_2_ particles under these conditions was −64.5 mV. Therefore, it is expected that a certain amount of PDDA can adsorb on the surface of the SiO_2_ particles and maintain a sufficient positive charge. The applied voltage and duration of the deposition were fixed at 7 V and 8 min, respectively. When the quantity of PDDA added to the coating sol was very low (3.0 × 10^−3^ wt%), the SiO_2_ and CB particles precipitated in the coating sol, and cathodic EPD could not be carried out. On the other hand, homogeneous coating films can be obtained *via* cathodic EPD from coating sols containing greater than 5.9 × 10^−3^ wt% PDDA. In particular, a vivid green structural color was observed for the coating film prepared from the sol containing PDDA with a quantity of 5.9 × 10^−3^ wt%. After addition of PDDA, the zeta-potential of SiO_2_ particles was inverted to +43.4 mV. It is noteworthy that the green color vanished when an increased quantity of PDDA was added to the coating sols. The quantitative reflectance spectra of the cathodic EPD coating films prepared from the sols containing PDDA are shown in Fig. 2b. The quantity of PDDA was varied from 5.9 × 10^−3^ to 23.6 × 10^−3^ wt%. The strong peak around *ca.* 550 nm, which is ascribed to coherent light scattering from the amorphous array of SiO_2_ particles, is observed in all spectra. The overall magnitude of the reflectance increased with increasing quantities of PDDA. This originates from the incoherent light scattering across the entire visible region. CB can reduce the contribution of such incoherent scattering by absorbing light uniformly across the entire visible region and can enhance the structural color of the arrays of SiO_2_ particles. These results indicate that amount of CB incorporated into the coating films decreased with increasing quantities of PDDA. When a large quantity of PDDA was added to the sol, a relatively large difference arose between the SiO_2_ and CB particles in the adsorption amounts of PDDA. The SiO_2_ particles obtained a higher positive charge than the CB particles under these condition. As a result, the SiO_2_ particles were deposited preferentially on the cathode substrates, and the amount of CB incorporated into the films decreased. Based on these results, we determined that the optimal quantity of PDDA to add to the sols is 5.9 × 10^−3^ wt% and fixed this concentration for the following experiments.

### 3.2. Influence of the quantity of CB particles on film color

**Fig. 3:**
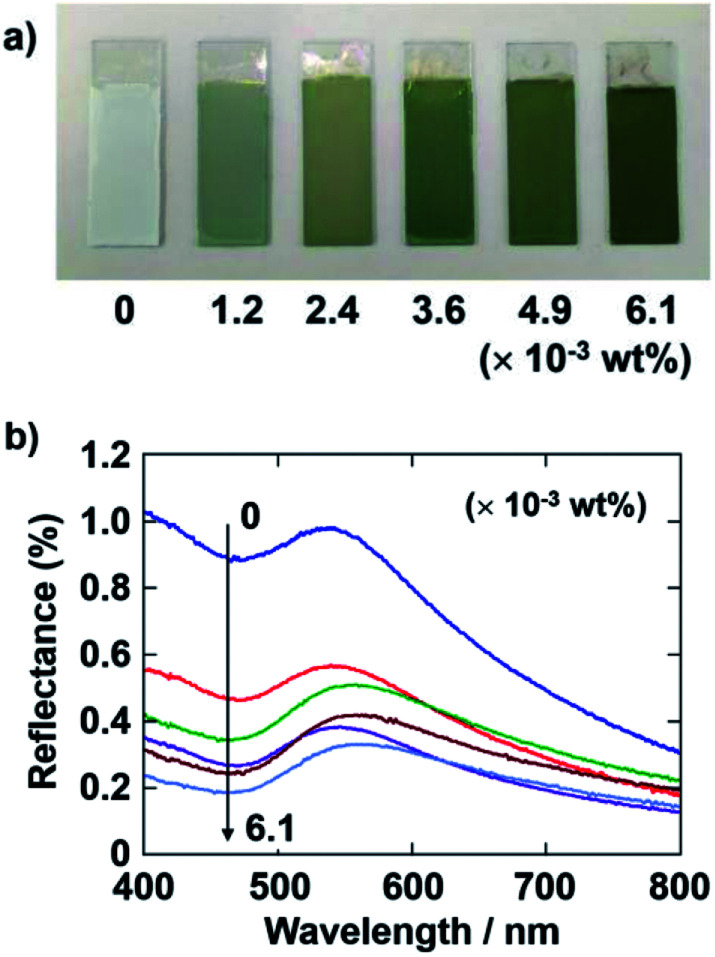
(a) Optical photographs of coating films prepared *via* cathodic EPD using PDDA. The quantity of CB added to the coating sol was varied from 0 to 6.1 × 10^−3^ wt%. SiO_2_ particles with a diameter of 260 nm. The quantity of PDDA in the coating sols was 5.9 × 10^−3^ wt%. The applied voltage and deposition time were 7 V and 8 min, respectively. (b) Reflectance spectra of the coating films shown in (a).

Next, the quantity of CB added to the coating sol was also varied at this fixed PDDA concentration. The applied voltage and deposition time of the EPD were fixed at 7 V and 8 min, respectively. Fig. 3a shows optical photographs of the EPD coating films obtained by varying the quantity of CB added. The coating film prepared without CB has a faint structural color and appears almost white to the naked eye. This is because of the strong incoherent light scattering. The coating films exhibit a bright green color when they were prepared with CB, which can reduce the contribution of incoherently scattered light. The color saturation of the coating films was found to greatly increase with CB incorporation. Quantitative reflectance spectra were obtained (Fig. 3b). In the spectrum of the film without CB, a peak due to the coherent light scattering and strong incoherent background due to multiple scattering are observed. In contrast, the overall magnitude of the reflectance greatly decreased with CB incorporation, whereas the intensity of the peak component seemed to be stable. Adding 3.6 × 10^−3^ wt% CB to the coating sol resulted in the most vivid green color of the EPD film. However, the color of the coating films darkened when additional CB, *i.e.*, greater than 4.9 × 10^−3^ wt%, was added to the coating sol. Therefore, we fixed the quantity of CB to add to the coating sols at 3.6 × 10^−3^ wt% for the following experiments.

### 3.3. Influence of the size of SiO_2_ particles on film color

**Fig. 4:**
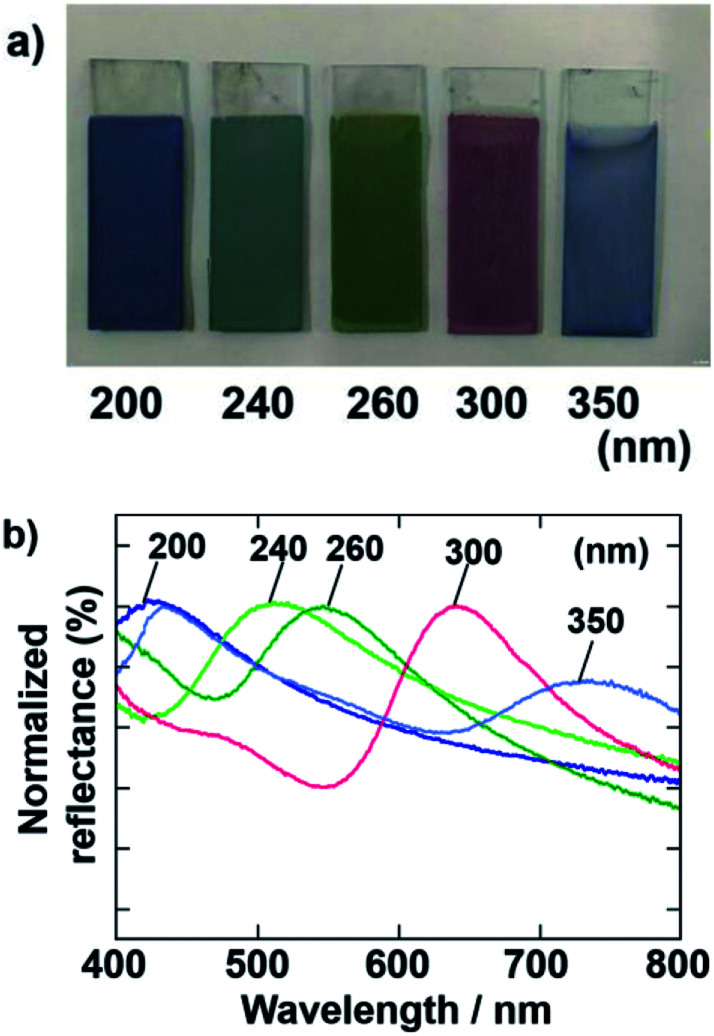
(a) Optical photographs of coating films prepared *via* cathodic EPD using PDDA. The size of SiO_2_ particles was varied from 200 to 350 nm. The quantities of PDDA and CB added to the coating sols were 5.9 × 10^−3^ and 3.6 × 10^−3^ wt%, respectively. The applied voltage and deposition time were 7 V and 8 min, respectively. (b) Normalized reflectance spectra of the coating films shown in (a) min, respectively.

Cathodic EPD coating films were also prepared by using SiO_2_ particles with various diameters. The applied voltage and deposition time of the EPD were fixed at 7 V and 8 min, respectively. Fig. 4 shows optical photographs and normalized reflectance spectra of the films prepared using SiO_2_ particles with diameters of 200, 240, 260, 300, and 350 nm. The color of the films was tunable by changing the diameter of the SiO_2_ particles used in the EPD process. In accordance with our previous report about anodic EPD coating films,^40^ blue, blue-green, green, and red coating films were prepared using different-sized particles. The peaks in the normalized reflectance spectra shift to longer wavelengths proportional to the SiO_2_ particle size.^33^ The sample prepared using SiO_2_ particles with a diameter of 350 nm has blue color. There are two peaks in the reflectance spectrum of this film (Fig. 4b). The peak due to the coherent light scattering exists around 750 nm. Usually, reflection in such near infrared region cannot be recognized to the human eyes. Another peak exists around 450 nm. Therefore, we can see the blue color from this sample. This peak does not due to the coherent light scattering, probably due to the Mie scattering.

### 3.4. Influences of EPD duration and applied voltage on the thickness of films

**Fig. 5:**
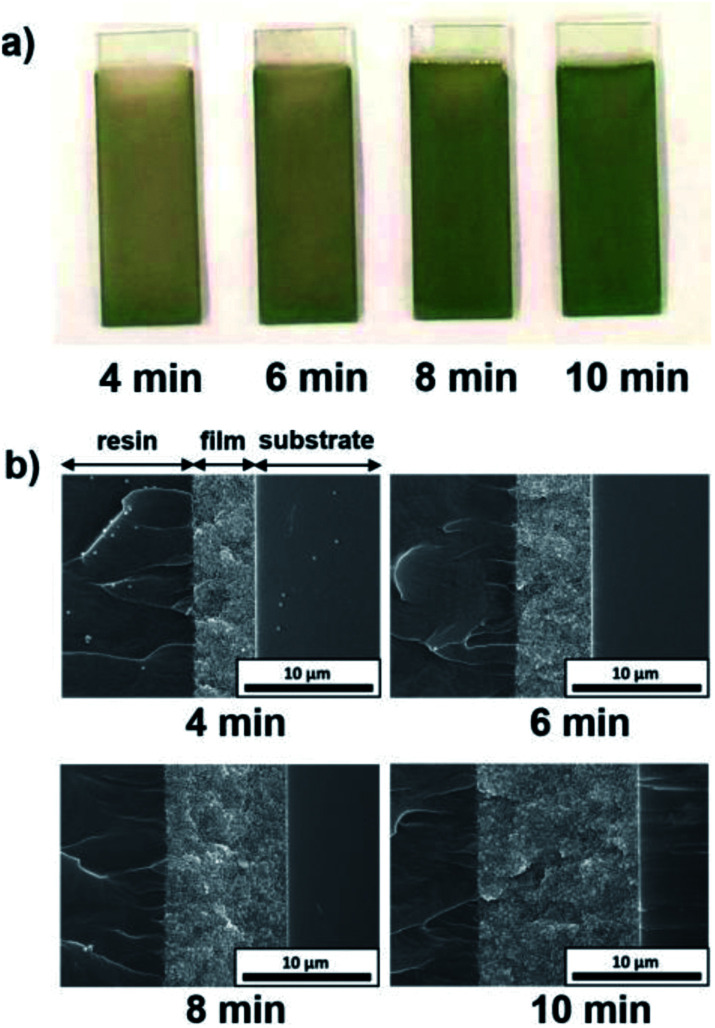
(a) Optical photographs and (b) cross-sectional SEM images of the cathodic EPD coating films prepared using SiO_2_ particles with a diameter of 260 nm on ITO-coated glass substrates. The quantities of PDDA and CB added to the coating sols were 5.9 × 10^−3^ and 3.6 × 10^−3^ wt%, respectively. The applied voltage was fixed at 5 V, and the deposition durations were 4, 6, 8, and 10 min. Samples for the cross-sectional SEM observation were fabricated by cutting coated films covered with epoxy resin.

**Fig. 6:**
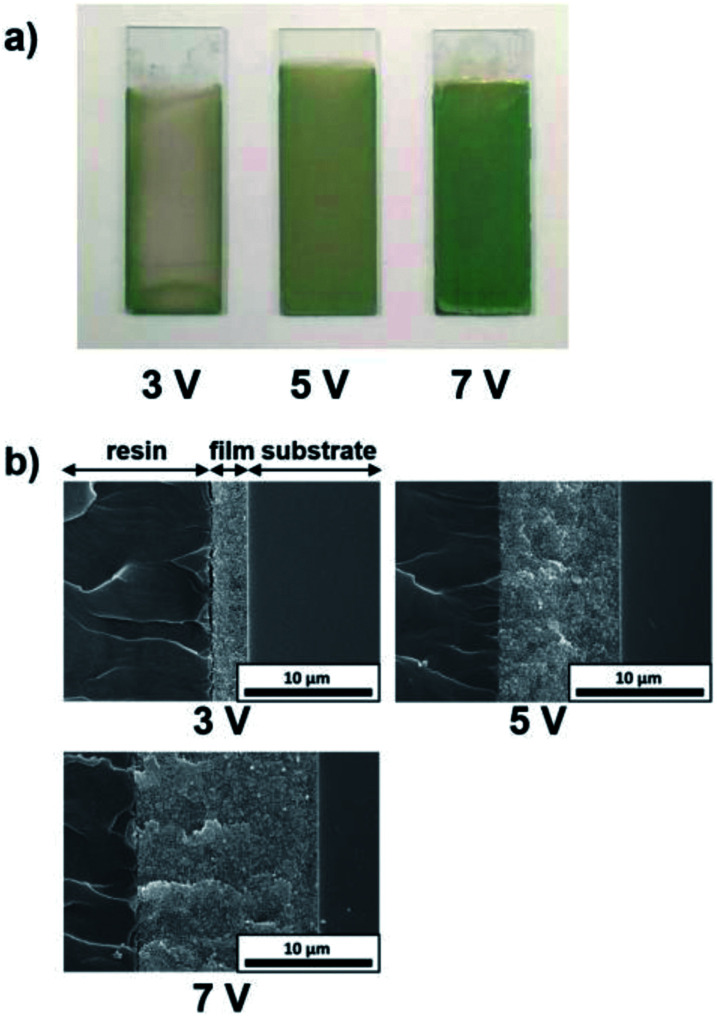
(a) Optical photographs and (b) cross-sectional SEM images of the cathodic EPD coating films prepared using SiO_2_ particles with a diameter of 260 nm on ITO-coated glass substrates. The quantities of PDDA and CB added to the coating sols were 5.9 × 10^−3^ and 3.6 × 10^−3^ wt%, respectively. The deposition duration was fixed for 8 min, and the applied voltages were 3, 5, and 7 V. Samples for the cross-sectional SEM observation were fabricated by cutting coated films covered with epoxy resin.

The influence of the EPD duration on the thickness of the coating films was examined. SiO_2_ particles with a diameter of 260 nm were employed, and the applied voltage was fixed at 5 V. The EPD duration was varied from 4 to 10 min. Optical photographs and cross-sectional SEM images of cathodic EPD coating films on ITO-coated glass substrates prepared with different deposition times are shown in Fig. 5. The coated films for observation by cross-sectional SEM were covered with epoxy resin before cutting to prevent mechanical damage. According to the cross-sectional SEM images, the morphology of the film is homogeneous in the direction of the depth. The interface between the coating film and the ITO-coated glass substrate indicates good contact. The coating film obtained *via* EPD for 4 min exhibits faint structural color. The thickness of the film is *ca.* 5 μm (Fig. 5b). It appears that insufficient coherent light scattering is generated from a film with such a thickness. The thickness of the films increases proportional to the EPD duration (Fig. S3 (ESI†)); *e.g.*, it reached *ca.* 10 μm for the film obtained *via* EPD for 8 min. A vivid green color was observed in this film. Next, the influence of the applied voltage on the thickness of the coating films was investigated. Here, the EPD duration was fixed at 8 min. The applied voltage was varied from 3 to 7 V. Fig. 6 shows optical photographs and cross-sectional SEM images of cathodic EPD coating films on ITO-coated glass substrates prepared with different applied voltages. The coating film obtained *via* EPD at 3 V is *ca.* 3 μm in thickness, and it exhibits faint structural color. On the other hand, the coating film prepared at 5 V has a thickness of *ca.* 10 μm and a vivid green structural color. The thickness of the films increases proportional to the applied voltage of the EPD process (Fig. S4 (ESI†)). Here, water electrolysis was not observed. A mixture of water and ethanol as a dispersant. The percentage of water is only 20 mol%. Therefore, water electrolysis can be suppressed even in the high voltage condition. These results indicate that in our cathodic EPD system, the thickness of the coating films can be controlled by both the EPD duration and applied voltage. In addition, film thicknesses greater than 10 μm are required to obtain brilliant structural color.

### 3.5. Influence of the pH of water added to the coating sols on the iridescence of the structural color of the EPD films

**Fig. 7:**
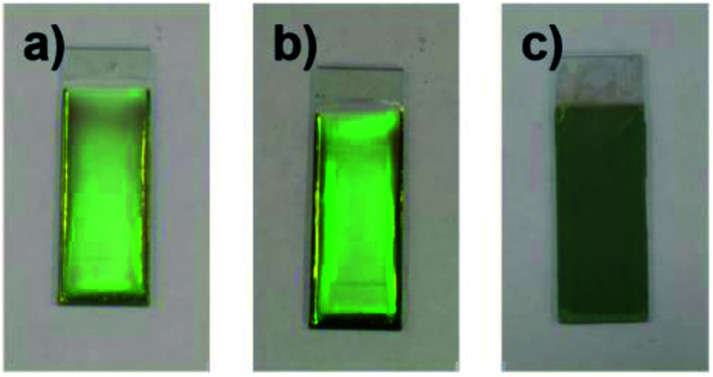
Optical photographs of coating films prepared *via* cathodic EPD using PDDA as a polycationic additive. The coating sols are under (a) acidic (pH 5.0), (b) neutral (deionized water), and (c) basic (pH 11.3) conditions. SiO_2_ particles with a diameter of 260 nm were used. The quantity of CB in the coating sols was 3.6 × 10^−3^ wt%. The quantity of PDDA was 5.9 × 10^−3^ wt%.

The influence of the pH of the water added to the coating sol on the arrangement of the colloidal array of SiO_2_ particles prepared *via* cathodic EPD was also investigated. Fig. 7 shows optical photographs of the cathodic EPD coating films obtained from coating sols prepared with water at various pH.

**Fig. 8:**
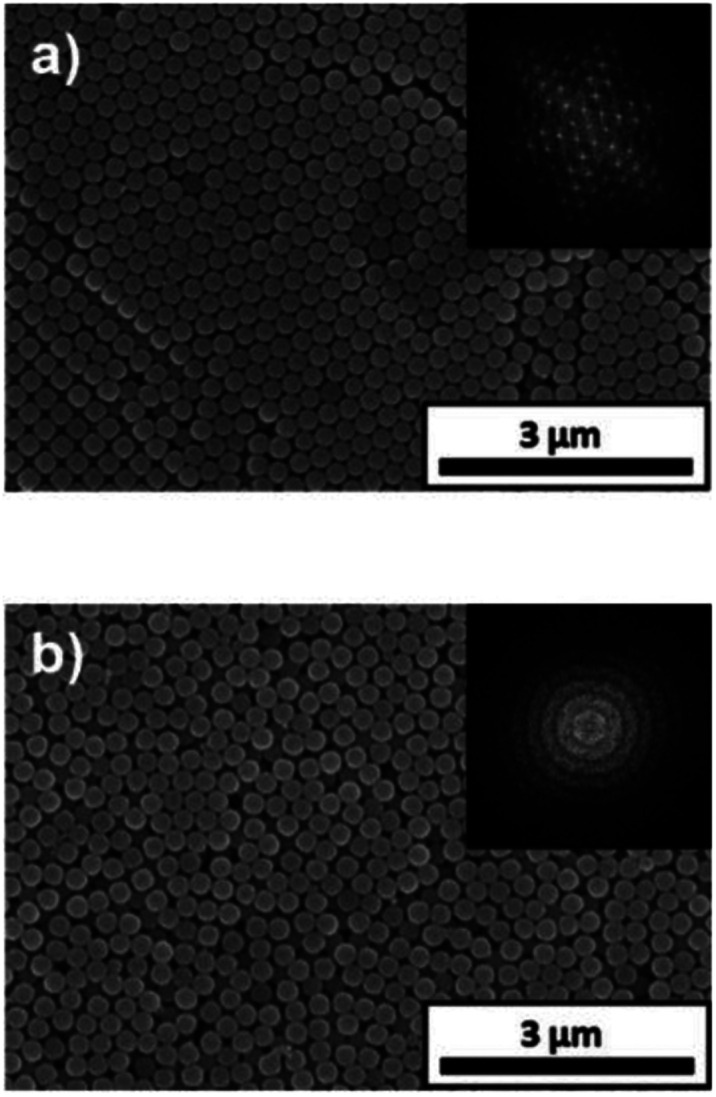
Surface SEM images of EPD coating films prepared using SiO_2_ particles with a diameter of 260 nm on ITO-coated glass substrates. The coating sols were prepared with water under (a) neutral (deionized water) and (b) basic (pH 11.3) conditions. The quantities of PDDA and CB added to the coating sols were 5.9 × 10^−3^ and 3.6 × 10^−3^ wt%, respectively. The applied voltage was fixed at 7 V. The EPD durations were 8 and 3 min for neutral and basic pH conditions, respectively. 2D Fourier power spectra from the SEM images are given as insets.

**Fig. 9:**
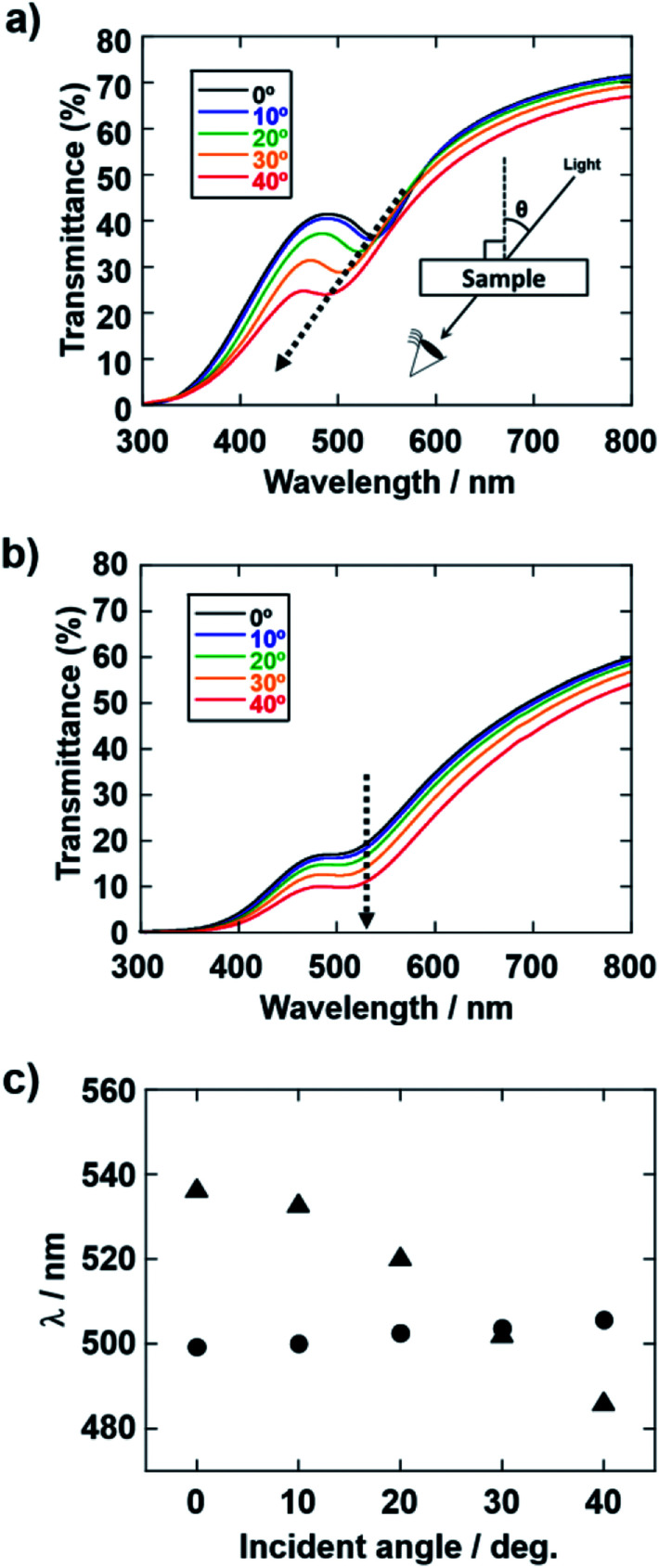
(a and b) Transmission spectra of the coating films corresponding to changes in the incident angle. The coating films were prepared using SiO_2_ particles with a diameter of 260 nm on ITO-coated glass substrates. The quantities of PDDA and CB added to the coating sols were 5.9 × 10^−3^ and 3.6 × 10^−3^ wt%, respectively. The applied voltage was fixed at 7 V. The EPD durations were 3 and 8 min. The coating sols were prepared with water under (a) neutral (deionized water) and (b) basic (pH 11.3) conditions. The incident angles were 0, 10, 20, 30 and 40° relative to the normal of the planar surface of the coating films. (c) Plots of the valley wavelength (*λ*) in the transmission spectra of the coating films as a function of the incident angle. Triangle, film prepared under neutral conditions; circle, film prepared under basic conditions.

**Fig. 10:**
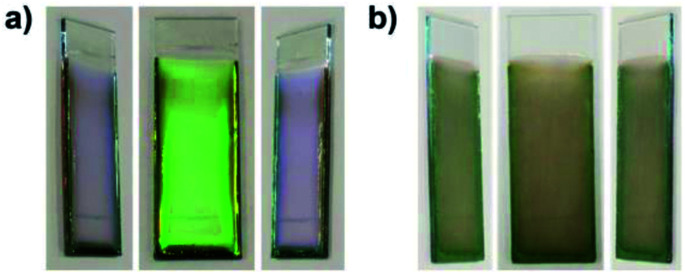
Optical photographs of the EPD coating film observed from various viewing angles. The coating films were prepared using SiO_2_ particles with a diameter of 260 nm on ITO-coated glass substrates. The quantities of PDDA and CB added to the coating sols were 5.9 × 10^−3^ and 3.6 × 10^−3^ wt%, respectively. The applied voltage was fixed at 7 V. The EPD durations were 8 and 3 min for neutral and basic pH conditions, respectively. The coating sols were prepared with water under (a) neutral (deionized water) and (b) basic (pH 11.3) conditions.

The applied voltage and deposition time were fixed to 7 V and 8 min, respectively. SiO_2_ and CB particles were well-dispersed even in the presence of PDDA at any pH. The formation of homogeneous coating films can be confirmed from each of the images. These films exhibit green structural color originating from the coherent light scattering of the arrays of SiO_2_ particles with a diameter of 260 nm. Interestingly, iridescent structural color was obtained from the coating sols under acidic and neutral conditions (Fig. 7a and b), whereas non-iridescent, matte structural color was obtained from the coating sol at basic pH (Fig. 7c). These results indicate that both iridescent and non-iridescent structurally colored coating films can be prepared from the coating sols by changing only the pH. The origin of the difference in the iridescence of the structural color is attributable to the difference in the arrangement of the SiO_2_ particles in the coating films. We have carried out independent measurements of the ordering of these coating films using SEM combined with an image processing technique, *i.e.*, two-dimensional (2D) fast Fourier transform (FFT), to determine the periodicity of the SiO_2_ particle arrangement. Fig. 8 shows surface SEM images of cathodic EPD coating films prepared from coating sols under neutral and basic pH conditions. 2D-Fourier power spectra from the SEM images are also given in the insets. The applied voltage was fixed at 7 V. The EPD duration was 3 and 8 min for basic and neutral pH conditions, respectively. A close-packed arrangement with long-range order appeared in the image of a coating film prepared at neutral pH (Fig. 8a), and sharp hexagonal patterns appeared in the corresponding 2D FFT image (inset of Fig. 8a). On the other hand, a disordered arrangement (without long-range order) was confirmed from the image of a coating film prepared under basic pH conditions (Fig. 8b), and only a broad circular pattern appeared in the corresponding 2D FFT image (inset of Fig. 8b). This observation indicates that the microstructure of the coating film is isotropic and has short-range order. In the case of basic pH conditions, the degree of dissociation of the Si–OH groups on the surface of the SiO_2_ particles is relatively high. Therefore, a large amount of PDDA deposited on the particles, and the particles obtained a strong positive charge. As a result, the electrophoresis rate of the particles toward the cathode is fast, and the deposited particles tightly adhere to the cathode. In this case, the rearrangement of particles after deposition is prevented, and a colloidal amorphous array is generated. This mechanism is analogous to that for the formation of a colloidal amorphous array *via* anodic EPD with high applied voltage in our previous work.^40^ In contrast, the amount of PDDA deposited on SiO_2_ particles at neutral pH seems to be much lower than that at basic pH. In this case, the attractive force between the particles and the cathode is weak, so the electrophoresis rate of the SiO_2_ particles should be slow. SiO_2_ particles tend to rearrange after their deposition on the cathode surface. As a result of the rearrangement, a colloidal crystal array is obtained under neutral conditions. This mechanism is also analogous to that for the formation of a colloidal crystal *via* anodic EPD with low applied voltage in our previous work.^40^ To quantify the angular dependence of the structural color, we characterized the angle-resolved optical properties of the coating films. Fig. 9a and b and show transmission spectra of coating films prepared *via* cathodic EPD at neutral and basic pH, respectively (the same as the films presented in Fig. 8). The incident angle was varied from 0° to 40°. The relationship between the valley wavelengths in the transmission spectra and the incident angles is also shown in Fig. 9c. In the transmission spectra, the valley position correlates with the color of the coating film. The valley position (*λ*) for the coating films prepared at neutral pH appeared at 537 nm when the incident angle was 0° and was shifted to 487 nm when the incident angle was 40°. This large shift in *λ* can be attributed to the fact that the structural color was based on Bragg reflection from the periodic structure of the coating film shown in Fig. 8a. In the case of coating films prepared at basic pH, the position of *λ* shifted from 500 nm to 506 nm when the incident angle was varied from 0° to 40°. The shift in *λ* in this range is negligible compared with that of the coating film prepared at neutral pH. The optical photographs of these coating films were taken at different angles from the direction of incident light (Fig. 10). In the case of the coating film prepared at neutral pH, the color of the film varied with the viewing angle. A blue color is observed from the side-view image of the coating film, whereas the brilliant green color is observed when the image was taken from the front. On the other hand, in the case of the coating film prepared at basic pH, the color of the film is almost constant even when viewed from directions different from the direction of irradiation light. These results indicate that the structurally colored coating films with and without iridescence can be fabricated controllably by varying the pH condition of our cathodic EPD system. Basic pH conditions are preferable to obtain a colloidal amorphous structure and non-iridescent structural color, whereas neutral pH conditions are preferable to obtain a colloidal crystalline structure and iridescent structural color.

### 3.6. Abrasion-resistant properties of structurally colored coating films

**Fig. 11:**
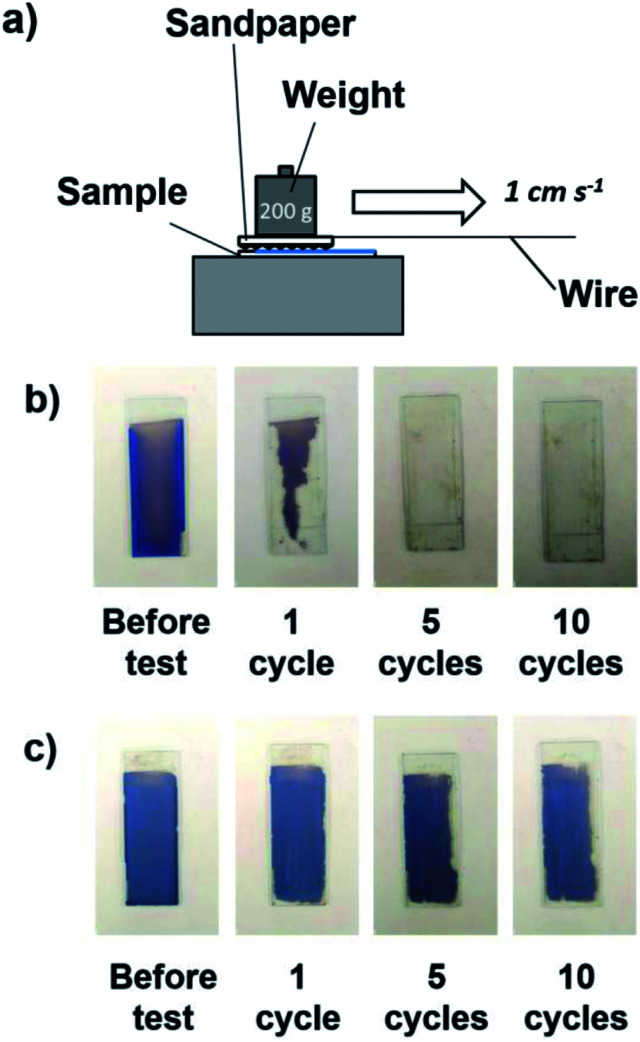
(a) Schematic of the sandpaper abrasion test carried out on the coating film on a glass substrate. (b and c) Optical photographs of the EPD coating films prepared *via* (b) anodic EPD and (c) cathodic EPD using PDDA before and after the sandpaper abrasion test (1, 5, and 10 cycles). The coating films were prepared using SiO_2_ particles with a diameter of 200 nm on ITO-coated glass substrates. In the case of anodic EPD, the applied voltage and EPD duration were 50 V and 2 min, respectively. In the case of cathodic EPD, the applied voltage and EPD duration were 7 V and 8 min, respectively.

The sandpaper abrasion test was reported to be an effective route to evaluate the mechanical abrasion resistance of coating films. Similar to a previously reported method,^53–55^ a sample with the coating was placed against sandpaper (#600 mesh) and weighted with 200 g of weight. The sample was moved 3 cm in a straight manner as shown in Fig. 11a. This was denoted a cycle, and a total of 10 cycles were conducted. The coating films were prepared by cathodic EPD using PDDA at 7 V for 8 min. For comparison, the abrasion resistance of structurally colored coating films prepared *via* the anodic EPD process, which was reported in our previous paper,^40^ was also investigated. In the case of anodic EPD, a coating sol without a polycationic additive was used, and the applied voltage and EPD duration were 50 V and 2 min, respectively. As shown in Fig. 11b, the coating film prepared *via* the anodic EPD process began to be abraded (nearly 50%) by the sandpaper after only one cycle and was completely scratched off after 5 cycles, which showed the weak adhesion between the coating and the substrate without adhesive. In contrast, nearly 90% of the coating films (in area) prepared *via* the cathodic EPD process using PDDA remained even after 10 cycles of abrasion testing (Fig. 11c). These results indicate that PDDA played important roles not only in endowing positive charges the SiO_2_ (and CB) particles with positive charge but also in serving as a binder to adhere particles to the substrate and with each other. PDDA has positive charges, whereas SiO_2_ particles have negative charges. Therefore, PDDA can prevent the electrostatic repulsion between SiO_2_ particles each other.

## 4. Conclusions

In summary, we have demonstrated for the first time that structurally colored coating films composed of arrays of monodisperse SiO_2_ particles can be successfully prepared by the cathodic EPD process. PDDA was an optimal polycation to obtain homogenous structural coating films *via* our cathodic EPD system. This is because that PDDA is a strong polycation and because the degree of dissociation of the ionic groups is nearly pH independent over a wide pH range. An optimal concentration of PDDA to obtain vivid structural color also exists for cathodic EPD. Precipitation of the SiO_2_ and CB particles occurred when a lower amount of PDDA was added to the coating sols. On the other hand, the structural color of the coating films vanished when large amount of PDDA was added to coating sols because the amount of CB incorporated into the coating films decreased upon increasing the amount of PDDA in the coating sol. Various vividly colored coatings can be produced from SiO_2_ particles with diameters between 200 and 350 nm. The thickness of the coating films can be controlled by varying the applied voltage and/or deposition time as well as the anodic EPD system. The arrangement of the particles in the array and the iridescence of the resultant structural color can be controlled by varying the pH of the coating sols for cathodic EPD. This interaction between SiO_2_ and PDDA is influenced by the pH. When the coating films are prepared at neutral pH, a close-packed array of SiO_2_ particles that exhibits an iridescent structural color is obtained. In contrast, an amorphous packing state can be acquired from coating sols under basic pH conditions. The structural color generated from such coating films has a low angular dependence. Generally, it is very difficult to control the iridescence of the structural color of coating films by conventional techniques such as spray coating. Therefore, it is noteworthy that the cathodic EPD system developed here can achieve controllable fabrication of structurally colored coating films with and without iridescence only by varying the pH of the coating sols. Moreover, PDDA acts as a binder, providing better adhesion of the SiO_2_ particles with each other and to the substrate. The coating film prepared by cathodic EPD displayed abrasion resistance superior to that prepared by anodic EPD. Nearly 90% of the coating films remained even after 10 cycles of abrasion using sandpaper. The ability to rapidly fabricate structurally colored coatings with controlled iridescence and abrasion resistance opens the door to the design of paints for decorations on a variety of surfaces, ranging from small everyday items to large surfaces, such as traffic signs, displays, and automobile bodies.

## Conflicts of interest

There are no conflicts to declare.

## Supplementary Material

RA-008-C8RA01215F-s001
