# Supplementary material for: Structurally colored coating films with tunable iridescence fabricated via cathodic electrophoretic deposition of silica particles
Source: RSC Adv. 2018 Mar 16;8(20):10776–84. doi: 10.1039/c8ra01215f (PMC9078918; doi:10.1039/c8ra01215f)
Supplement: RA-008-C8RA01215F-s001 [file RA-008-C8RA01215F-s001.pdf]

Electronic Supplementary Material (ESI) for RSC Advances.
This journal is © The Royal Society of Chemistry 2018

Electronic Supplementary Information (ESI)

Structurally colored coating films with tunable iridescence fabricated via cathodic electrophoretic deposition of silica particles

Kiyofumi Katagiri,*[a] Kensuke Uemura,[a] Ryo Uesugi,[a] Kei Inumaru,[a] Takahiro Seki[b] and Yukikazu Takeoka*[b]

[a] *Department of Applied Chemistry, Graduate School of Engineering, Hiroshima University, 1-4-1 Kagamiyama, Higashi-Hiroshima 739-8527, Japan. E-mail: kktgr@hiroshima-u.ac.jp.*

[b].*Department of Molecular and Macromolecular Chemistry, Graduate School of Engineering, Furo-cho, Chikusa-ku, Nagoya 464-8603, Japan. E-mail: ytakeoka@apchem.nagoya-u.ac.jp*

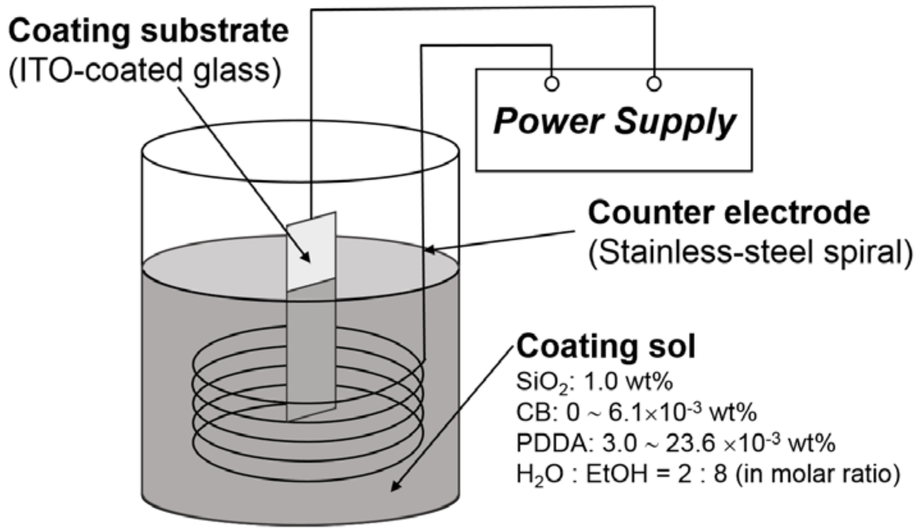

Fig. S1 Schematic drawing of the setup of the EPD system used in the present study.

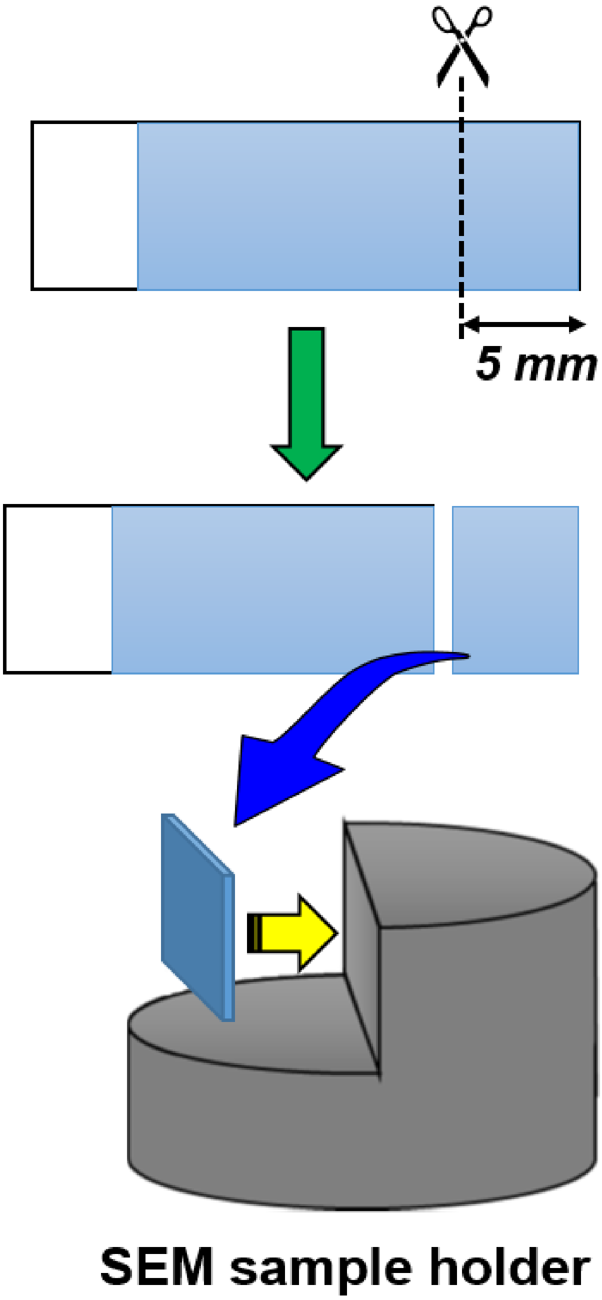

Fig. S2 Schematic drawings of the procedure for cross-sectional SEM observation of an EPD coating film consisting of a SiO_2 particle array.

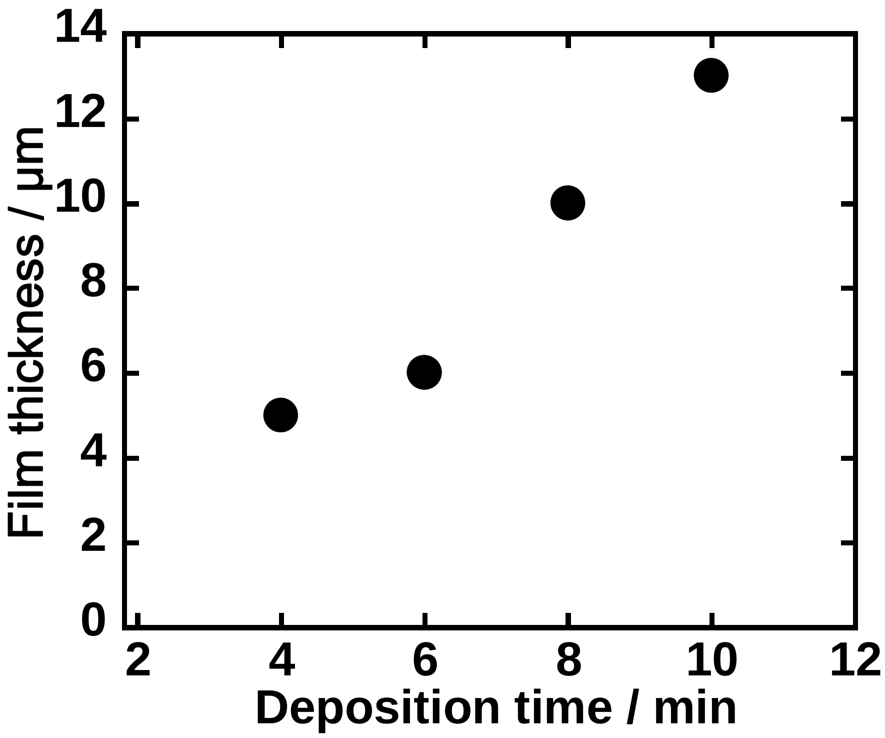

Fig. S3 Thickness of the cathodic EPD coating films as a function of the EPD duration. The coating films were prepared using SiO_2 particles with a diameter of 260 nm on ITO-coated glass substrates. The quantities of PDDA and CB added to the coating sols were 5.9×10^{-3} and 3.6×10^{-3} wt%, respectively. The applied voltage was fixed at 5 V.

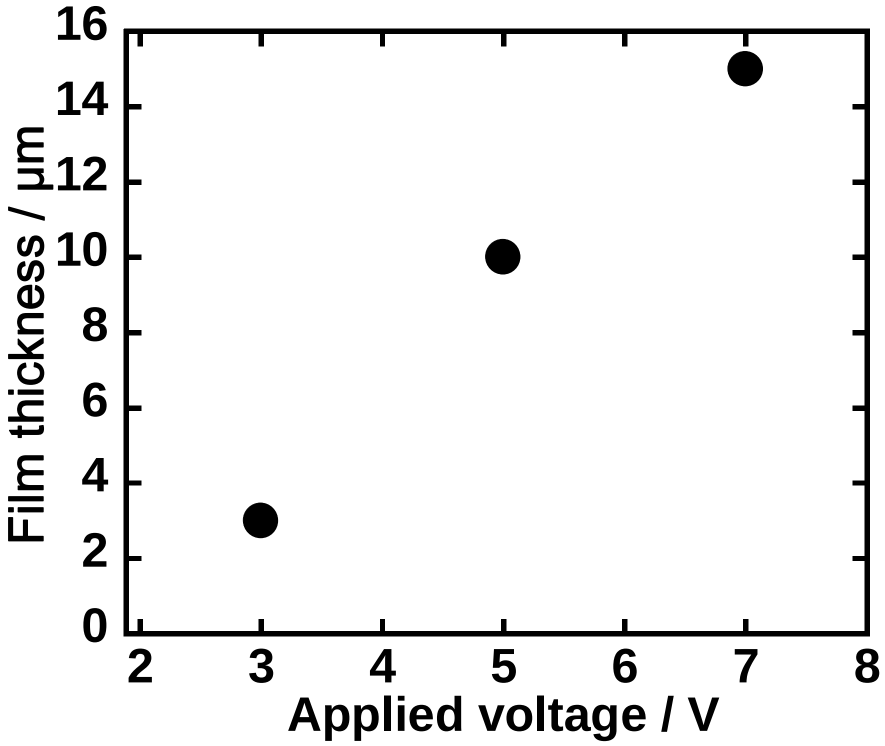

Fig. S4 Thickness of the cathodic EPD coating films as a function of applied voltage of EPD process. The coating films were prepared using SiO_2 particles with a diameter of 260 nm on ITO-coated glass substrates. The quantities of PDDA and CB added to the coating sols were 5.9×10^{-3} and 3.6×10^{-3} wt%, respectively. The EPD duration was fixed at 8 min.
